# The Multifaceted Roles of EGFL7 in Cancer and Drug Resistance

**DOI:** 10.3390/cancers13051014

**Published:** 2021-03-01

**Authors:** Beate Heissig, Yousef Salama, Satoshi Takahashi, Ko Okumura, Koichi Hattori

**Affiliations:** 1 Department of Immunological Diagnosis, Graduate School of Medicine, Juntendo University School of Medicine, 2-1-1 Hongo, Bunkyo-Ku, Tokyo 113-8421, Japan; kokumura@juntendo.ac.jp; 2 An-Najah Center for Cancer and Stem Cell Research, Faculty of Medicine and Health Sciences, An-Najah National University, P.O. Box 7, Nablus 99900800, Palestine; yousef.ut@najah.edu; 3 Division of Molecular Therapy, Center for Stem Cell Biology and Regenerative Medicine, The Institute of Medical Science, The University of Tokyo, 4-6-1 Shirokanedai, Minato-ku, Tokyo 108-8639, Japan; radius@ims.u-tokyo.ac.jp; 4 Center for Genome and Regenerative Medicine, Graduate School of Medicine, Juntendo University, 2-1-1 Hongo, Bunkyo-Ku, Tokyo 113-8421, Japan; khattori@juntendo.ac.jp

**Keywords:** beta 3 integrin, integrin, cancer, drug resistance, angiocrine factor, angiogenesis, EGFR, EGFL7, miR-126, adhesion, migration, protease, EMT, FAK, LOX, KLF2, ECM, endothelial cells, cancer, proliferation

## Abstract

**Simple Summary:**

Cancer growth and metastasis require interactions with the extracellular matrix (ECM), which is home to many biomolecules that support the formation of new vessels and cancer growth. One of these biomolecules is epidermal growth factor-like protein-7 (EGFL7). EGFL7 alters cellular adhesion to the ECM and migratory behavior of tumor and immune cells contributing to tumor metastasis. EGFL7 is engaged in the formation of new vessels and changes in ECM stiffness. One of its binding partners on the endothelial and cancer cell surface is beta 3 integrin. Beta 3 integrin pathways are under intense investigation in search of new therapies to kill cancer cells. All these properties enable EGFL7 to contribute to drug resistance. In this review, we give insight into recent studies on EGFL7 and its engagement with beta3 integrin, a marker predicting cancer stem cells and drug resistance.

**Abstract:**

Invasion of cancer cells into surrounding tissue and the vasculature is an important step for tumor progression and the establishment of distant metastasis. The extracellular matrix (ECM) is home to many biomolecules that support new vessel formation and cancer growth. Endothelial cells release growth factors such as epidermal growth factor-like protein-7 (EGFL7), which contributes to the formation of the tumor vasculature. The signaling axis formed by EGFL7 and one of its receptors, beta 3 integrin, has emerged as a key mediator in the regulation of tumor metastasis and drug resistance. Here we summarize recent studies on the role of the ECM-linked angiocrine factor EGFL7 in primary tumor growth, neoangiogenesis, tumor metastasis by enhancing epithelial-mesenchymal transition, alterations in ECM rigidity, and drug resistance. We discuss its role in cellular adhesion and migration, vascular leakiness, and the anti-cancer response and provide background on its transcriptional regulation. Finally, we discuss its potential as a drug target as an anti-cancer strategy.

## 1. Introduction

Tumor growth and metastasis rely on the tumor vascular network for adequate delivery of oxygen and nutrients [1]. Tumor endothelial cells (ECs) are the cellular building blocks of the nutrient-carrying vasculature. During tumor growth, activated ECs expand and form new capillaries in a process called angiogenesis [2]. These capillaries and vessels carry nutrients to hungry cancer cells and ensure proper oxygen delivery.

ECs release so-called angiocrine factors [3], which include the angiogenic factor vascular endothelial growth factor-A (VEGF-A), Jagged1 (Jag1), endothelin, enzymes such as tissue-type plasminogen activator [3], and epidermal growth factor-like protein-7 (EGFL7) [4]. EGFL7 is produced by cancer-associated ECs [4,5] and certain tumor cell types [4,6].

EGFL7 controls intercellular and cell–matrix communication, which are key features of tumor progression and metastasis, by hijacking the receptor tyrosine kinase epidermal growth factor receptor (EGFR), integrin, and Notch signaling pathways [6,7,8].

EGFL7 modulates cell migration by interacting with extracellular matrix (ECM) sensing integrins [9]. Integrins are a family of cell surface receptors that help cells to interact with the extracellular microenvironment, thereby controlling cell anchorage and movement. Integrins exist as heterodimers with noncovalently linked alpha and beta subunits and link the cytoskeleton with the ECM [10]. Integrins are mechanotransducers and key factors during cell migration and are thereby implicated in many steps of cancer progression, starting with primary tumor development to metastasis, cancer stem cell development, and drug resistance (reviewed in [11]). EGFL7 interacts with two of the most studied integrins in cancer—namely, alphaV:beta 3 (ITGAV:ITGB3) and the alpha5:beta1 integrin (ITGA5:ITGB1).

Integrins bind to a wide range of ECM proteins containing the arginylglycylaspartic acid (RGD)-motif. EGFL7 is one of those ECM proteins with a conserved RGD/Glutamine-Glycine-Asparagine (QGD) motif [12]. The RGD motif is exposed once EGFL7 attaches to the ECM but is hidden in the soluble form of EGFL7 [13]. The ITGAV:ITGB3 integrin can bind to fibronectin, collagen, fibrinogen, thrombospondin, and EGFL7, among others [7]. EGFL7 with its RGD motif competes for binding to ITGAV:ITGB3 integrin with matrix metalloproteinase2 (MMP2), fibronectin, and collagen IV. ITGB3 has important roles in angiogenesis, tumor metastasis, and drug resistance, leading to the development of novel specific RGD-like ligands for use in anti-tumor therapy (reviewed in [14]).

In this review, we introduce the angiocrine factor EGFL7 and one of its receptors (ITGB3) as regulators of angiogenesis and summarize recent knowledge on their involvement in tumor metastasis. We also discuss their involvement in drug resistance in cancer.

## 2. Epidermal Growth Factor-Like Protein-7

**Figure 1:**
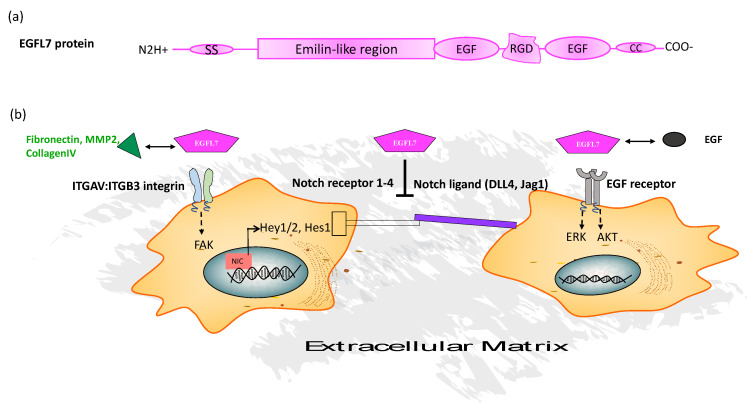
(**a**) Model structure of the EGFL7 protein. EGFL7 contains two EGF-like repeats: an arg-gly-asp integrin-binding motif (RGD) and an Emilin-like region. (**b**) The regulatory network of EGLF7. EGFL7 binds via its RGD domain to ITGAV:ITGB3 integrin and causes among others FAK autophosphorylation. EGFL7 competes for binding to the integrin with the ECM molecules fibronectin, MMP2, and collagen IV. The Emilin-like region of EGFL7 interacts with Notch receptor 1–4 and Notch ligands DLL4 and Jag1 and suppresses Notch signaling, resulting in impaired NIC translocation into the nucleus and reduced Hey1/2 and Hes1 transcription. EGFL7 binding to EGFR results in the activation of the signaling pathways extracellular signal-regulated kinase (ERK) and AKT, among others. EGFL7 competes for binding to EGFR with EGF. Abbreviations: EGF, epidermal growth factor; EGFL7, epidermal growth factor-like protein 7; EGFR, epidermal growth factor receptor; ERK, extracellular signal-regulated kinase; FAK, focal adhesion kinase); MMP2, matrix metalloproteinase 2; DLL4, delta like-protein 4; Jag1, Jagged1; NIC, Notch intracellular domain; ECM, extracellular matrix.

Mouse and human EGFL7 were cloned in 2003 by Soncin [5]. EGFL7 is a molecule that contains an N-terminal signaling sequence, followed by a cysteine-rich Emilin-like (EMI) domain and two epidermal growth factor-like (EGF-like) domains [5] (Figure 1a). The microRNA-126 gene (miR126) is located within intron 7 of the EGFL7 gene. Studies on the effects of miR126 in tumorigenesis are not covered in this review.

The EGFL7 gene locus contains binding sites for the transcription factors Krüppel-like factor 2 (KLF2) [15] and SMAD1/5 [16,17]. EGFL7 expression was upregulated on ECs by the blood-flow-sensitive transcription factor KLF2a after ITGB1-mediated induction [18] and by SMAD transcription factors after the binding of bone morphogenic protein-9 to the transmembrane anaplastic lymphoma kinase 1 receptor [16,17].

Whilst high expression is found during embryonic and neonatal development [19], EGFL7 is downregulated in almost all mature tissues except in the adult mouse lung, with lower expression in the heart, ovary, uterus, and kidneys [20]. EGFL7 expression rises again during vascular injury [21], during pregnancy, in regenerating endothelium following arterial injury, in growth plate injury [12], in atherosclerotic plaques, and in growing tumors, often mainly in tumor ECs [4].

**Figure 2:**
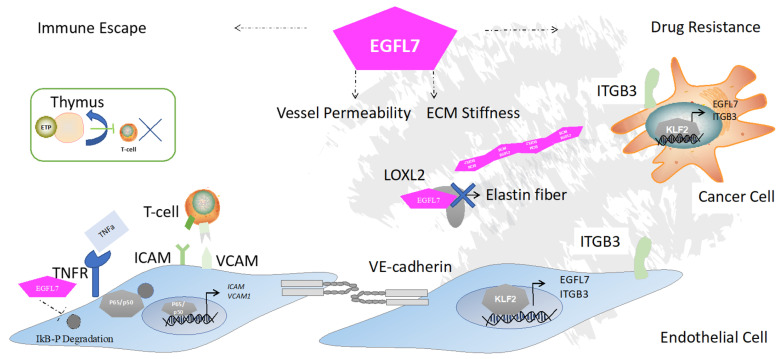
EGFL7 alters tumor growth and metastasis by suppressing the production of immune cells and their recruitment into the growing tumors, vessel permeability, ECM stiffness—all of which contribute to drug resistance. T cell adhesion and rolling and transmigration through the EC are required for T cells to cross the vascular barrier. EGFL7 allows the tumor to escape from the anti-tumor immune response by preventing terminal T cell differentiation in the thymus and inhibiting T cell recruitment via suppression of the adhesion molecules ICAM and VCAM on ECs. EGFL7 prevents adhesion molecule transcription after tumor necrosis factor alpha (TNFa) stimulation by blocking Nuclear factor kappa B (NFkB) signaling. EGFL7 controls ECM stiffness by interacting with LOXL2 so as to mitigate covalent crosslinking of collagen or elastin. ITGB1 integrin on ECs and _ITGB3_ integrin on cancer cells [45] induce the expression of transcription factor KLF2 which enhances EGFL7 expression, resulting in enhanced cell proliferation. Myelosuppressive drugs such as bortezomib were shown to enhance KLF2-mediated upregulation of ITGB3 and EGFL7. Abbreviations: KLF2, Krüppel-like factor 2; ETP, early thymic progenitor; LOXL2, lysyl oxidase-like 2.

EGFL7 is a 41-kDa secreted signaling factor [4,5] that can be deposited into the ECM. EGFL7 contains a positive C- and a negative N-terminus, enabling the formation of EGFL7 oligomers that are deposited in the ECM in a head-to-tail fashion (Figure 2). It was recently shown that docking of the EGFL7 protein into both fibers and individual aggregates of the EC extracellular space requires the microfibrillar component microfibrillar-associated glycoprotein-1 and fibronectin [17]. The study demonstrated that docking of EGFL7 to the ECM is required for its effects on lysyl oxidase (LOX) activity, but that ECM binding was not necessary to mediate its effect on endothelial adhesion molecule expression or Hairy/enhancer-of-split related with YRPW motif protein 2 (Hey2) expression along the Notch pathway (Figure 1).

EGFL7 facilitates angiogenesis and tumorigenesis. It stimulates the recruitment and proliferation of embryonic or human brain ECs [7,22] and primary mouse embryonic fibroblasts [21]. EGFL7, through its multiple binding partners and cellular receptors (reviewed in [23]), can be found on various cell types, including tumor cells and ECs (Figure 1b). EGFL7 can bind to the NOTCH1–4 extracellular domain through its Emilin-like region [24]. EGFL7 competes with the Notch ligands Jag1 and Jag2 for Notch binding and inhibits Notch signaling (Figure 1b). EGFL7 competes with the Notch ligand Delta-like-4 for Notch4 binding on ECs, while suppressing Notch downstream signals like Hey1 and Hairy/enhancer-of-split 1 (Hes1) and promotes angiogenesis [22]. In acute myeloid leukemia, a hematopoietic blood cell cancer, recombinant EGFL7 inhibited DELTA-like 4-mediated Notch activation while anti-EGFL7 in combination with Dll4 increased Notch activation and induced apoptosis. 

EGFL7 can also bind to the EGF receptor on the cell membrane, which results in the activation of the signaling pathways mitogen-activated protein kinase/extracellular signal-regulated kinase (ERK) and phosphatidylinositol-3-kinase/AKT [6,25] (Figure 1b). It was reported that EGFL7 binds to EGFR wildtype but not to the active mutant EGFR variant III, leading to b-catenin activation and upregulation of EGFL7 expression and tumor growth [26]. Binding of EGFL7 to EGFR enhanced cell migration of hepatocellular carcinoma cells and increased intrahepatic and pulmonary metastases in murine liver cancer models but did not alter tumor cell proliferation [27]. On the cellular level, EGFL7–EGFR binding caused phosphorylation of the cytoplasmic protein focal adhesion kinase (FAK) [27]. It is interesting to note that EGFR is expressed on intratumoral vessels but not vessels in non-tumor tissues [28,29,30,31,32], which would suggest that EGFL7 binding to EGFR could drive tumor-angiogenesis. But studies so far indicate that the EGFL7-driven pro-angiogenic effects are mainly mediated by Notch receptor or integrin and not by EGFR signaling [16,33].

EGFL7 enhanced migration of the siman virus 40-mouse microvascular endothelial (SVEC) cell line and resulted in the phosphorylation of ERK1/2, FAK, and STAT5 [12]. EGFL7 treatment of EGFL7-induced SVEC migration was blocked in the presence of RGD peptides, demonstrating the involvement of integrin signaling in EC migration. FAK is activated upon integrin or growth factor receptor signaling, resulting in the autophosphorylation at tyrosine (Y) 397. FAK is a key mediator of integrin signaling through its association with focal adhesion proteins, such as paxillin and talin. The role of FAK as both a cytosol and nuclear protein contributing to cancer progression has been recently reviewed by Murphey et al. [34].

Integrins mediate cell adhesion to the ECM. Adhesions serve as traction points and as signaling centers during cell migration [35]. There is an optimal strength of attachment that allows sufficient adhesion for traction at the cell front and yet allows for efficient release at the rear [36]. Integrin activation in protrusions regulates actin polymerization and myosin II activity through Rho-family GTPases such as Cdc42, Rac1, and RhoA [37]. EGFL7 potentiates EC migration on fibronectin-coated plates through binding to ITGAV:ITGB3 integrin [7], resulting in the activation of the downstream target GTPase Cdc42. EGFL7 cannot directly bind to ITGA5:ITGB1 integrin but enhanced angiogenesis involving this integrin [13,38]. Mechanistically, EGFL7 binding to ITGAV:ITGB3 integrin blocked the endocytosis of fibronectin-associated ITGA5:ITGB1 integrin [13] and ITGAV:ITGB3 and resulted in the upregulation of both integrins on the EC surface, allowing focal adhesion maturation, hydrolysis of Rac1-GFP, and enhanced migration speed of ECs on fibronectin surfaces [39]. 

EGFL7 controls proliferation in melanoma, hepatocellular carcinoma, and clear cell renal cell carcinoma [40,41,42] through one of its receptors. Blood cell cancers such as acute myeloid leukemia (AML) or the plasma cell malignancy multiple myeloma (MM) have dysfunctional integrin and Notch signaling [43,44,45]. EGFL7 caused acute myeloid leukemia (AML) blast proliferation [46]. Anti-EGFL7 blocking antibody through reactivation of Notch signaling in AML cells induced cell differentiation and apoptosis in vitro and in vivo [46]. Our group demonstrated that malignant plasma cells from patients with MM adhere to ECM-deposited EGFL7 and that their cell growth and survival required EGFL7 binding to ITGB3 [45]. These studies demonstrate that EGFL7 could be a potential cancer target.

## 3. EGFL7 Contributes to the Pathological Tumor Vessel Phenotype

As one of the hallmarks of cancer, angiogenesis is necessary for the transition of a small, localized tumor with a diameter of around 1–2 mm into an invasive disease. EGFL7 contributes to the branched and disorganized architecture of tumor endothelium with irregular multi-layered EC lining and an inconsistent smooth muscle and pericyte sheath.

Sprouting angiogenesis refers to the de novo formation of new vessels via the local proliferation of and extension of ECs from the wall of an existing vessel [47]. Cellular components of newly formed vessels include tip cells, which migrate in response to gradients of EGFL7 and VEGF, stalk cells, which proliferate and extend the vessels, and phalanx cells, which are quiescent and support the sprout [48]. For the formation of vessels, EC migration requires the coordinated attachment to and de-attachment from the surrounding ECM [36]. EGFL7 is a component of the interstitial ECM deposited mainly on the basal sides of sprouts at the interface between ECs and interstitial cells. The EGFL7 deposits form a unique ECM coat on the sprout surface. This coat outlines the boundary of a new sprout and marks the migratory path of new ECs [13]. In EGFL7-deficient ECs, the lack of EGFL7-mediated scaffolding of the ECM leaves ECs clueless on where to move. ECs not only attach in the path of the sprout but also attach to the basal sides of other ECs, leading to larger sprouts with multiple layers of ECs on top of each other. The accumulation of ECs hinders appropriate EC movement. Therefore, EGFL7 contributes to the hallmark excessively branched and disorganized architecture of tumor endothelium. All these features from EGFL7 contribute to an unstable vessel wall and promote the vessel leakiness that is characteristic of tumor ECs. Tumor EC-derived EGFL7 promotes glioma growth in experimental glioma models and stimulates tumor vascularization with the generation of mature vessels covered with pericytes and smooth muscle cells [13]. A recent study demonstrated that treatment of ECs with melanoma cell-derived exosome enhanced VE-Cadherin, uPAR, and EGFR upregulation in ECs [49], resulting in a tumor EC phenotype, and through EGFR gave EGFL7 a chance to promote neoangiogenesis.

During the metastatic stage of intravasation, tumor cells gain access to the circulation via vascular or lymphatic vessels, enabled by the transiently increased permeability of the tumor vasculature. Vascular impermeability is achieved with a barrier comprised of ECM components such as the endothelial glycocalyx, the endothelium, basement membrane, and accessory cells such as pericytes and smooth muscle cells. Besides vessel-wrapping, pericytes and smooth muscle cells ensure that no fluid, protein, or immune cell leakage occurs from blood to tissue [50]. Tumor vessels show inconsistent pericyte or smooth muscle cell coverage [51]. EGFL7 reduces EC smooth muscle coverage by blocking platelet-derived growth factor-BB-mediated smooth muscle cell migration [5]. Tumor vessels have irregular cell surface cell walls and show leakiness. EGFL7 knockdown in ECs showed a disturbance of adherens junctions with insufficient phosphorylation of VE-cadherin that contributed to increased EC permeability [33] (Figure 2). EGFL7 knockdown suppresses VEGF-A-mediated angiogenesis, causes the overproduction of endothelial filopodia on the basal side of ECs, and reduces collagen IV deposition at the basal side of ECs. These data demonstrate that EGFL7 controls the actions of one of its angiocrine allies, VEGF. VEGF has been shown to enhance vessel permeability by a cross-activation of ITGB3 and VEGFR2 that directly regulates VE-cadherin [52]. 

## 4. EGFL7 Enhances Tumoral Immune Escape

Immune cells, such as T lymphocytes, are part of the body’s weapons to fight off tumor cells through their capacity for antigen-directed cytotoxicity. EGFL7 compromises the anti-tumor response on two levels: it impairs the production of terminally differentiated T lymphocytes and it prevents lymphocytes from crossing the vasculature into the tumor bed.

The Flt3/Flt3-ligand pathway is important for early thymic precursors (ETP) expansion [53] and lymphocyte (T and B cell) development. EGFL7 blocks terminal T cell differentiation but expands ETPs in the thymus, the place where T cell precursors reside [54]. Mechanistically, EGFL7-mediated suppression of Notch signaling [55] enhanced the fms-related tyrosine kinase 3 (Flt3) promoter activity in ETPs, causing upregulation of FLT3 on ETPs. EGFL7 also increased the number of thymic ECs, which are a source of Flt3 ligand. Together, EGFL7 stimulates Flt3/Flt3 ligand signaling in ETPs that resulted in the accumulation of immature ETPs and a paucity of circulating T cells and contributes to impaired antigen-directed cytotoxicity against tumor cells.

At the center for the anti-tumor response of immune cells is the ability of immune cells to reach the tumor tissues. Leukocyte immune cell traffic out of the bloodstream into the tumor tissues requires the activation of adhesion molecules. ECs can actively impact T lymphocyte migration by changing the expression of leukocyte adhesion molecules on their surface. The activation of very late antigen-4 (VLA-4; ITGA4:ITGB1, CD49d/CD29) and lymphocyte function-associated antigen 1 (LFA-1,αLβ2, CD11a/CD18) integrin by talin and kindlin allow firm interaction between the immune cell-like T cells or neutrophils and ECs, which express integrin ligands such as intercellular adhesion molecules (ICAMs), vascular cell adhesion molecule 1 (VCAM1), and MAdCAM (reviewed in [56]). These molecules enable T lymphocytes to adhere to ECs (Figure 2), a process that is required for them to cross into the tumor bed. Inhibiting leukocyte migration into the tumor niche at the EC level represents one way for cancer cells to escape anti-tumor host immune responses.

Following the initial observation that EGFL7-overexpressing tumor cells showed enhanced tumor growth and metastasis in immunocompetent but not in immunodeficient mice with an impaired influx of inflammatory cells [57], it was shown that EGFL7 modulates the immune cell recruitment process. Mechanistically, EGFL7 downregulated the endothelial adhesion molecules ICAM1 and VCAM1 on the ECs of 4T1 breast cancer and LLC1 lung adenocarcinoma tumors [57]. Follow-up studies revealed that the treatment of ECs with tumor necrosis factor-alpha (TNFa) repressed VCAM1 or ICAM1 expression and downregulated ICAM1 and VCAM1 transcription via an NFkB dependent mechanism [58]. Because a lack of adhesion factors prevents lymphocytes from binding to ECs, a necessary step for cells to cross the vasculature and enter the tumor bed, EGFL7-induced alteration of adhesive properties endows tumors with the ability to escape immune attack [57] (Figure 2).

## 5. EGFL7 Regulates ECM Stiffness and EMT

Fibroblasts and tumor cells produce large quantities of the ECM molecules’ collagen and fibronectin [59]. The generated highly fibrotic tumor microenvironment causes ECM stiffness that favors tumor progression/metastasis and epithelial–mesenchymal transition (EMT) [60,61]. The lysyl oxidase family comprising LOX and four lysyl oxidase-like proteins (LOXL1–4) (reviewed recently [62]) contributes to ECM stiffness by catalyzing the covalent cross-linking of collagen and elastin. Pro-LOX is synthesized and secreted as a pro-enzyme and, following procollagen and C-proteinase cleavage, gives rise to LOX-PP and LOX. LOX-PP interacts with collagen I, LOXL2, MMP-2, fibromodulin, EGF [63], and as a recent study demonstrated, with EGFL7 [64]. Indeed, EGFL7 binds to all members of the LOX family including LOX and LOXL1–4 [64,65], indicating its importance in the regulation of tumor ECM stiffness. Earlier studies demonstrated that EGFL7 suppresses LOXL2 function [65]. EGFL7 binding to the catalytic LOXL2 domain impaired the conversion of tropoelastin into mature insoluble elastin [65] (Figure 2).

High ECM stiffness promotes EMT and metastasis [60]. In the early stages of metastasis, epithelial cells decrease the expression of cell–cell junction molecules at the primary tumor site and become motile [66,67]. The upregulation of the EMT-associated genes Twist and Snail and the loss of the epithelial marker E-cadherin and the induction of the mesenchymal marker vimentin are typical gene expression patterns during EMT [68]. It was shown that EGFL7 promoted metastasis by triggering EMT in gastric cancer cells with the downregulation of E-cadherin and upregulation of vimentin and Snail [6]. 

## 6. EGFL7 Contributes to Drug Resistance

Many cancer patients who receive neoadjuvant or adjuvant treatment to surgery relapse months or years later with a tumor that is resistant to further chemotherapeutic challenge, a feature known as chemoresistance [69]. Despite advances in cancer treatment and the initial tumor reduction after drug treatment, cancer cells develop drug resistance. Resistance to anti-cancer treatment is dependent not only on genetic mutations and epigenetics but also on external factors [70], including cytokines, growth factors, enzymes, glycoproteins, extracellular vesicles, angiocrine factors, and integrins. We showed that irradiation augmented EGFL7 expression in thymic ECs [54]. Drug resistance to the proteosome inhibitor and anti-myeloma bortezomib often occurs in MM patients. Our group reported that treatment with bortezomib, but not other drugs, induced EGFL7 expression in MM cells [45] (Figure 2).

Integrin/ECM interactions mediate cell adhesion-mediated drug resistance, activating a pro-survival and anti-apoptotic program. This escape strategy of tumors is either due to the survival of cells already expressing certain integrins and/or cells capable of inducing integrin gene expression (for review [70]). MM treatment using bortezomib upregulated ITGB3 on MM cells [45]. Our data indicated that targeting EGFL7 using neutralizing antibodies or ITGB3 using an integrin inhibitor could override drug resistance against bortezomib in vitro and using murine in vivo MM models. Mechanistically, we showed that EGFL7-mediated activation of ITGB3 induced the expression of transcription factor KLF2 that further augmented EGFL7 expression in MM cells. Our study demonstrated that an EGFL7-ITGB3-KLF2-EGFL7 amplification loop supports MM cell survival and proliferation in vitro as well as in vivo [45] (Figure 2). In other cell types, such as the human osteosarcoma, where EGFL7 is highly expressed in tumor cells even more so than in ECs, chemotherapy reduced EGFL7 expression [71]. These data suggest that each tumor cell type, dependent on the needs of the ECM niche, develops its own drug escape mechanism.

The existence of tumor cancer stem cells is another tumor and drug escape mechanism. Tumor-initiating cells represent a small fraction within the tumor. These cells have stem cell characteristics with the capacity for self-renewal, they rest in a non-cycling/quiescent state, and retain their ability to differentiate. First shown in neuronal stem cells, EGFL7 can modulate stem cell fate through its liaison with Notch receptors and ligands [24]. We showed that EGFL7 expands immature early thymic progenitor cells [54]. Since many anti-cancer drugs require tumor cell cycling, keeping tumor cells in a quiescent state is just another trick by which EGFL7 enhances drug resistance.

Integrin signaling functions depend on the cytoplasmic protein tyrosine kinase FAK, the major protein found in focal adhesions. While ITGB3-mediated tumor growth was thought to require downstream activation of FAK and cell adhesion, a recent study challenged this doctrine and showed that neither of them was required for c-Src activation and Crk-associated substrate phosphorylation that enhanced tumor growth [72]. Tavora et al. demonstrated that targeting of EC FAK sensitized tumors to DNA-damaging therapy [73]. EC FAK is necessary for the DNA damage-induced NFkB activation required for the cytokine production in ECs. Given that EGFL7 binding to ITGB3 can activate FAK in ECs [7,74], and EC FAK contributes to drug resistance, it is conceivable that blocking EGFL7-ITGB3 can improve chemosensitization to DNA-damaging therapies through the loss of FAK (reviewed in [75]).

## 7. Conclusions

The ECM molecule EGFL7 is a critical player in the metastatic program, an inhibitor of the anti-cancer immune response escape, and contributes to drug resistance. The effects of EGFL7 on tumor cells and ECs and its diversity of binding receptors enable EGFL7 to impinge on tumor survival strategies.

Proper signals from the ECM maintain stem cell fate. Certain integrins are exclusively abundant in epithelial stem cells, with one of them being ITGAV:ITGB3 integrin, which has been described on lung, breast, and pancreatic tumors with a stem-like phenotype [76]. A deeper understanding of the role of EGFL7 in controlling the fate of tumor-initiating cells and cancer cells and the identification of new ECM binding partners or signaling receptors will open up new avenues for cancer treatment. 

EGFL7 unites with main drivers of tumorigenesis from EGFR, ITGB3, Notch ligand, and receptors or LOX family members. Although anti-EGFL7 therapy alone could not sufficiently control tumor growth, the current state of research indicates that combining anti-EGFL7 therapy with other anti-cancer strategies such as chemotherapy might improve the efficacy of conventional anti-cancer strategies.
